# CAR T-Cell Therapy: Is CD28-CAR Heterodimerization Its Achilles’ Heel?

**DOI:** 10.3389/fimmu.2021.766220

**Published:** 2021-11-17

**Authors:** Leonardo M. R. Ferreira, Yannick D. Muller

**Affiliations:** ^1^ Department of Microbiology and Immunology, Medical University of South Carolina, Charleston, SC, United States; ^2^ Hollings Cancer Center, Medical University of South Carolina, Charleston, SC, United States; ^3^ Division of Immunology and Allergy, Department of Medicine, Lausanne University Hospital and University of Lausanne, Lausanne, Switzerland

**Keywords:** chimeric antigen receptor, CD28, CD19 CAR T-cell, transmembrane domain, heterodimerization, cytokine release syndrome, neurotoxicity, immune effector cell-associated neurotoxicity syndrome

## Introduction

Chimeric antigen receptor (CAR) T-cell therapy has dramatically expanded the success rate of cancer immunotherapy, especially in CD19-expressing blood cancers. Yet, it has also given rise to new complications, notably cytokine release syndrome, neurotoxicity, and, sometimes, fatal cerebral edema. The exact mechanisms of such toxicities across different CD19 CAR T-cell products, however, remain hotly debated. It was recently demonstrated that CARs containing a CD28 transmembrane domain (TMD) can heterodimerize with the endogenous CD28 receptor. Here, we hypothesize that, upon on-target activation, this heterodimerization is responsible for the increased sensitivity of CD19 CAR to CD19^low^ brain mural cells, resulting in increased risk of developing severe neurotoxicity. This hypothesis may only be confirmed with a clinical trial comparing two CD19-CD28-TMD CARs differing only by targeted amino-acid mutations in the CD28 transmembrane domain.

**Figure 1:**
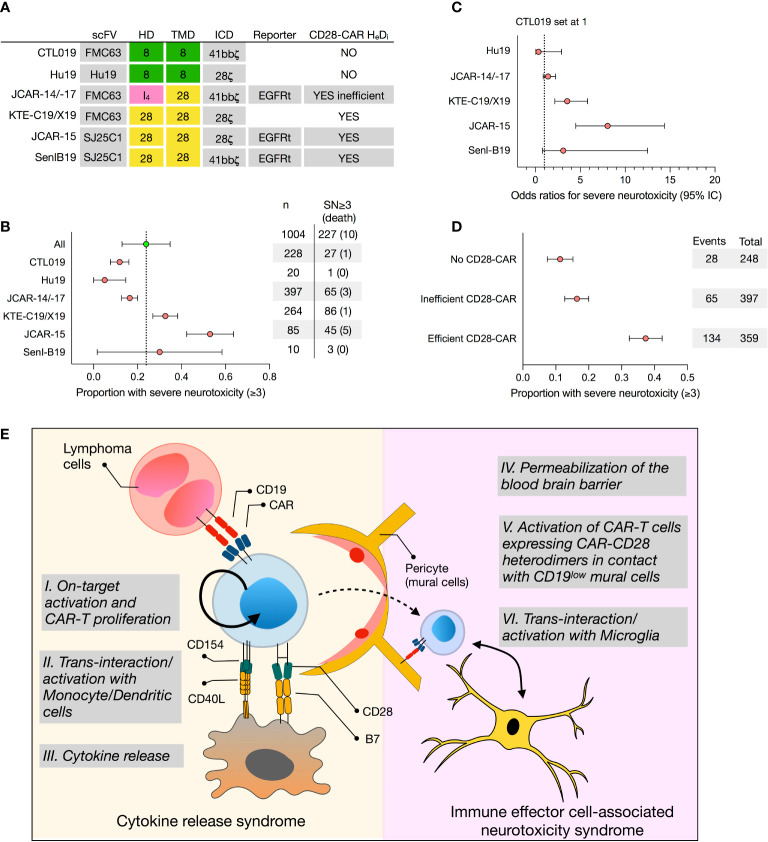
Retrospective analysis of the proportion of severe neurotoxicity of selected CD19 CAR T-cell products and proposed model for CAR T cell-mediated neurotoxicity. **(A)** Construct designs of 5 selected CD19 CAR T-cell products, namely tisagenlecleucel (CTL019), Hu19, JCAR-14/-17, axicabtagene ciluleucel (KTE-C19), JCAR-15, and SenI-B19, differing by their hinge (HD) and transmembrane (TMD) domain. **(B)** Forest plot representing untransformed proportions of severe neurotoxicities (SN, grade 3 or higher) among patients treated with CAR T-cell products. Confidence intervals (95%) were calculated using binary random effect and DerSimonian-Laird methods with OpenMeta (http://www.cebm.brown.edu/openmeta/index.html). **(C)** The odds ratios of grade 3 or higher severe neurotoxicity comparing Hu19, JCAR14-/17, KTE-C19, JCAR15, and Senl B19 CAR-T products with CTL019 (set as reference) are shown. Calculations were made on SPSS Statistics (IBM, New York, NY) and based on a Pearson Chi-Square test and logistic regression tests assuming that clinical monitoring among the different studies and CD19 CAR-T-cell product is comparable. **(D)** Forest plot representing untransformed proportions of severe neurotoxicities comparing CARs with no CD28-CAR heterodimers (Hu19, CTL019), inefficiently formed CD28-CAR heterodimers (JCAR-14/17), and efficiently formed CD28-CAR heterodimers (SenIB19, JCAR-15, KTE C19). **(E)** CAR T cells, following on-target activation (I.), undergo several rounds of proliferation in the absence of antigen. This proliferation, fueled by CD40L-CD40 and B7-CD28 interactions with monocytes and/or dendritic cells (II.), ultimately results in cytokine release syndrome (CRS) (III.). In turn, CRS compromises the blood-brain barrier (IV.), allowing CAR T cells to penetrate the central nervous system (CNS). If CAR-CD28 heterodimers assemble on the cell surface, CAR T cells in the CNS interact with mural cells expressing low levels of CD19 (V.), as well as with microglia expressing co-stimulatory receptors (VI.), triggering immune effector cell-associated neurotoxicity syndrome (ICANS). *H_e_D_i_, heterodimerization; SN, severe neurotoxicity; HD, hinge domain; TMD, transmembrane domain; ICD, intracellular domain*.

T lymphocytes engineered with anti-CD19 chimeric antigen receptors (CAR) are emerging as powerful treatments for leukemia and lymphoma. The US Food and Drug Administration (FDA) approved two CD19 CAR T-cell products in 2017, which have shown clinical efficacy in the treatment of relapsed/refractory (r/r) acute lymphoblastic leukemia (ALL) and r/r non-Hodgkin lymphoma (NHL). The first CAR product, tisagenlecleucel (KYMRIAH/Novartis Pharmaceuticals Corp., thereafter referred to as CTL019), originally developed by CAR T-cell pioneer Carl June and colleagues, is currently approved for patients up to 25 years of age with r/r ALL and, since 2018, for adults with r/r NHL. In 2017, axicabtagene ciloleucel (YESCARTA/Kite Pharma, Inc., a Gilead Sciences Company, thereafter referred to as KTE-C19), is approved for adult patients with r/r NHL. Since then, two other CD19-CAR T-cell products have been FDA-approved: brexucabtagene autoleucel in 2020 (KTE-C19/TECARTUS/Kite Pharma, Inc., thereafter referred to as KTE-X19, a product differing only from KTE-C19 by an extra-step in the manufacturing process to exclude malignant circulating cells) for adult patients with r/r mantle cell lymphoma, and in 2021 lisocabtagene maraleucel (BREYANZI/Juno Therapeutics, Inc., a Bristol-Myers Squibb Company, thereafter referred to as JCAR-17, a product with the same CAR design as its previous generation JCAR-14) for adult patients with r/r large B-cell lymphoma. Notably, these CAR-T have the same single chain variable fragment (scFv), but different hinge (HD), transmembrane (TMD), and intracellular signaling domains (ICD) (**Figure 1A**).

## Safety Concerns of CAR T-Cell Therapy

Although CAR T-cell therapy can induce spectacular clinical remission, safety remains an important concern with up to one-third of the patients developing significant toxicities, namely cytokine release syndrome (CRS) and immune effector cell-associated neurotoxicity syndrome (ICANS) (1, 2). By 2018, eighteen patients died after receiving CD19-CAR T-cells (3). CRS is the most commonly observed cause of toxicity coinciding with the peak of CAR T-cell expansion (4), manifesting as fever, life-threatening hemodynamic instability with multi-organ failure, and, in some cases, fulminant hemophagocytic lymphohistiocytosis. ICANS is the second most common adverse event in CAR T-cell therapy ranging from mild cognitive impairment to an encephalopathic state characterized by confusion, delirium, seizures, and cerebral edema. ICANS can happen concurrently with or independently of CRS, a feature distinct from other organ-specific toxicities (1). The management of CRS and ICANS is currently based on administering anti-IL-6 monoclonal antibodies, sometimes together with corticosteroids. The latter are, however, avoided whenever possible to prevent inhibition of the infused CAR T cells (3). Importantly, ICANS normally resolves within 2-3 weeks after CAR T-cell infusion, although later recurrences are possible (3).

Notably, some CD19-CAR T cells products are more frequently associated with the development of severe ICANS (**Figure 1** and **Supplementary Table 1** and references therein). To address the rate of neurotoxicity among selected CD19 CAR-T cells products, we performed a linear regression analysis of reported severe neurotoxicity observed among 1004 patients treated with CTL019, Hu19, JCAR-14, JCAR-17, KTE-C19, KTE-X19, JCAR-15, and Senl-B19 (**Supplementary Table 1** and **Figures 1B, C**). The odds ratio of having grade 3 or higher severe neurotoxicity was significantly higher for KTE-C19 (3.5, 95% confidence interval (CI), 2.2-5.5) and JCAR-15 (8.0, 95% CI, 4.5-14.4) than with CTL019 (set to 1), JCAR-14/-17 (1.4 95%CI 0.9-2.3), Hu19 (0.3, 95% CI 0.05-2.9) and SenI-B19 (3.1, 0.8-12.5) (**Figures 1B, C**). These results are consistent with a recent meta-analysis (1). Additionally, they were also observed in a single clinical trial comparing side by side CAR T cells produced in the same conditions but engineered with a CAR design matching either CTL019 or KTE-C19 (5). Infusion with the KTE-C19-like product had to be suspended due to the high rate of neurotoxicity events (5). These data echo the unexpectedly high rate of severe ICANS 18/32, 56%) experienced during a phase 2 clinical trial, the ROCKET study, testing CD19-CAR T engineered with a CD28-HD, TMD and ICD (JCAR-15). This trial had to be terminated after the death of five patients from cerebral edema.

## Identifying CAR Features Associated With Toxicity

The mechanism behind the observed differences in CAR T-cell toxicity profiles between different products remains hotly debated. First, all main CAR T-cell products (accounting for >80% of infusions) share the same scFv, clone FMC63, ruling out major differences in CAR antigen affinity. Second, severe neurotoxicity was observed with CAR-T cells engineered with a CD28-ζ or 4-1BB-ζ ICD using lentiviral or retroviral transduction protocols (2, 6). Finally, no study found a link between the CD4/CD8 T cell ratio in the final CAR T-cell infusion product and neurotoxicity occurrence, even though the starting cell populations (PBMCs *vs.* enriched CD4 and CD8 T cells) and the expansion protocols (anti-CD3/CD28 beads *vs.* anti-CD3 alone) differed between them. Data from clinical studies show that tumor burden is a risk factor for developing CRS and ICANS (2). Recent preclinical studies showed that recipient’s monocytes can be transactivated *via* the CD40-CD40L pathway and responsible for the bulk of IL-1 and IL-6 production during CRS, excluding models based solely on the direct interplay between CAR T cells and tumor cells. Indeed, blocking IL-6 receptor with tocilizumab or using IL-1 receptor antagonist prevents CRS in mouse models, providing a rationale for using these monoclonal antibodies for the treatment of CRS after CAR T cell therapy (7). Another comprehensive analysis found a significant association between elevated pre-treatment disease burden and high peak CAR T-cell expansion, concomitantly with blood brain barrier disruption and central nervous system-specific production of IL-6, IL-8, MCP1, and IP10 (6). There was, however, no significant correlation between severe neurotoxicity and transfused CAR T-cell number or tumor cell presence in the brain. More recently, single-cell RNA sequencing surveys revealed the existence of rare (0.2% of brain cells) CD19-expressing cells in the brain: mural cells, including pericytes and vascular smooth muscle cells, which support vasculature and are critical for the integrity of the blood-brain barrier. This suggests that lysis of brain mural cells by CD19-CAR T cells may be partly responsible for ICANS (8).

Yet, those results do not explain why there is an increased risk of developing ICANS when infusing KTE-C19/KTE-X19 or JCAR-15 as compared to CTL019 (**Figures 1B, C**). Importantly, KTE-C19/KTE-X19 and JCAR-15 share the same hinge, transmembrane, and signaling domain, all derived from the CD28 molecule. It is known that CD28 signaling, as compared to 4-1BB, results in faster and larger magnitude changes in protein phosphorylation, influencing the response and differentiation of effector T cells (9). However, in a recent phase 1 clinical trial, Brudno et al. showed that a humanized CD19 CD28-zeta CAR containing a CD28 signaling domain but a CD8-derived hinge (HD) and transmembrane (TMD) domain resulted in much reduced severe neurotoxicity: only 5% of patients who received Hu19-CD8-CD28-zeta T cells (Hu19) experienced it versus 50% of patients who received KTE-C19 (10). On the other hand, Li and colleagues tested a CD19-CAR with a CD28-TMD/HD but a 4-1BB intracellular costimulatory domain (Senl-B19) and reported 30% of ICANS (11). While it must be acknowledged that both studies included only a limited number of patients, these results suggest that the CD28 signaling domain is not sufficient to provoke neurotoxicity and, more importantly, that the roles of the HD and TMD in CAR T-cell-mediated neurotoxicity are currently underestimated.

## The Impact of the CAR Transmembrane Domain in CAR T-Cell Toxicity

Several lines of evidence suggest that the CAR’s HD and TMD are not inert and can modulate CAR-T cell activation. Carl June and colleagues first showed that tonic signaling *via* CARs bearing a CD28-TMD, but not a CD8-TMD, sustained *in vitro* T-cell proliferation up to 3 months in the absence of exogenous IL-2 and following a single TCR stimulation (12). Alabanza et al. found that CD19-CAR T cells produced significant higher levels of inflammatory cytokines upon CD19 recognition if featuring a CD28-TMD/HD instead of a CD8-TMD/HD (13). Crystal Mackall and co-workers demonstrated that swapping the CD8-TMD/HD in a CD19 4-1BB-ζ CAR for a CD28-TMD-HD lowered the antigen density threshold for CAR T-cell activation (14). Finally, we have recently demonstrated that CD28 TMD-containing CARs can recruit and dimerize with endogenous CD28, which normally exists as a homodimer on the cell surface, *via* a four amino acid motif in the TMD (15, 16). Consistent with this, in-depth analysis of the CAR interactome and signalosome revealed that the top interacting partner of a CAR bearing a CD28-TMD/HD is endogenous CD28, and CAR mediated-signaling is associated with phosphorylation of endogenous CD28 (9, 17). This association, through heterodimerization of the CAR with endogenous CD28 receptor *via* the CD28-TMD (15), may result in stronger signal transduction, facilitating CAR T-cell activation in the context of low levels of CAR antigen, such as in low-CD19 mural cells. It is interesting to note that CD28-CAR heterodimerizes inefficiently if the CAR is built with an IgG4-HD. In silico modeling of the hinge-hinge interactions suggested that the membrane proximity of the IgG4 hinge is too short to form CAR-CD28 inter-molecular disulfide bonds for stabilizing the CAR-CD28 heterodimerization, leading to preferential CAR-homodimerization (15). This observation may explain why JCAR-14/-17, engineered with a CD28-TMD and IgG4-HD, caused less ICANS than KTE-C19/KTE-X19 or JCAR-15 (**Figures 1B, C**). The risk of developing ICANS may thus be directly linked to the capacity to form CD28-CAR heterodimers (**Figure 1D**).

## Discussion

In conclusion, we hypothesize that, while CAR T cells are specifically activated on-target, they will undergo several rounds of proliferation in the absence of antigen. This proliferation may be fueled by CD40L-CD40 and possibly also by CD28-B7 trans-interactions with monocytes and/or dendritic cells, ultimately resulting in CRS. This process may compromise the blood-brain barrier, facilitating the trafficking of CD19-CAR T cells into the central nervous system. Depending on whether CAR-CD28 heterodimers are efficiently formed and present on the cell surface, CAR T cells could interact with low-CD19 mural cells and with microglia, known to express co-stimulatory receptors, ultimately initiating ICANS (**Figure 1E**). The fitness of the cells as well as the level of CAR expression could directly influence the severity of neurotoxicity. It will be extremely challenging to validate this hypothesis based solely on preclinical mouse models. In our opinion, its best demonstration will come from a clinical trial comparing side by side CD19-CAR T cells differing only by select amino acid mutations in their TMD. Such results may have an important impact on the future design and choice of CD19-CAR T cells for hematological but also autoimmune disease treatment.

## Author Contributions

LF and YM wrote this manuscript. YM performed the meta-analysis. All authors contributed to the article and approved the submitted version.

## Funding

LF is an NIDDK Human Islet Research Network (HIRN) Emerging Leader in Type 1 Diabetes. YM is supported by a grant from the Gabriella Giorgi Cavaglieri foundation.

## Conflict of Interest

A provisional patent on CAR-CD28 heterodimerization has been submitted. The authors declare that the research was conducted in the absence of any other commercial or financial relationships that could be construed as a potential conflict of interest.

## Publisher’s Note

All claims expressed in this article are solely those of the authors and do not necessarily represent those of their affiliated organizations, or those of the publisher, the editors and the reviewers. Any product that may be evaluated in this article, or claim that may be made by its manufacturer, is not guaranteed or endorsed by the publisher.
